# A simple, portable, electrochemical biosensor to screen shellfish for *Vibrio parahaemolyticus*

**DOI:** 10.1186/s13568-017-0339-8

**Published:** 2017-02-15

**Authors:** Noordiana Nordin, Nor Azah Yusof, Jaafar Abdullah, Son Radu, Roozbeh Hushiarian

**Affiliations:** 1 Institute of Advanced Technology, Universiti Putra Malaysia (UPM), 43400 Serdang, Selangor Malaysia; 2 Food Safety Research Centre, Faculty of Food Science and Technology, Universiti Putra Malaysia, 43400 Serdang, Selangor Malaysia; 3 Department of Chemistry, Faculty of Science, Universiti Putra Malaysia (UPM), 43400 Serdang, Selangor Malaysia; 4 La Trobe Institute for Molecular Science, La Trobe University, Melbourne, VIC 3086 Australia; 5 Department of Life Sciences, Imperial College London, London, SW7 2AZ UK

**Keywords:** *Vibrio parahaemolyticus*, Food-borne pathogens, Electrochemical DNA sensor, Shellfish, Portable biosensor

## Abstract

An earlier electrochemical mechanism of DNA detection was adapted and specified for the detection of *Vibrio parahaemolyticus* in real samples. The reader, based on a screen printed carbon electrode, was modified with polylactide-stabilized gold nanoparticles and methylene blue was employed as the redox indicator. Detection was assessed using a microprocessor to measure current response under controlled potential. The fabricated sensor was able to specifically distinguish complementary, non-complementary and mismatched oligonucleotides. DNA was measured in the range of 2.0 × 10^−8^–2.0 × 10^−13^ M with a detection limit of 2.16 pM. The relative standard deviation for 6 replications of differential pulse voltammetry (DPV) measurement of 0.2 µM complementary DNA was 4.33%. Additionally, cross-reactivity studies against various other food-borne pathogens showed a reliably sensitive detection of the target pathogen. Successful identification of *Vibrio parahaemolyticus* (spiked and unspiked) in fresh cockles, combined with its simplicity and portability demonstrate the potential of the device as a practical screening tool.

## Introduction

*Vibrio parahaemolyticus (V. parahaemolyticus)* a gram-negative, halophilic bacterium is not only the leading cause of seafood-associated bacterial gastroenteritis in the United States (DePaola et al. 2000) but it is also one of the most important food-borne pathogens in Asia, causing around half of the foodborne outbreaks in Southeast Asian countries (Martinez-Urtaza et al. 2004). Additionally, it should be noted that the number of *V. parahaemolyticus* infections has increased and their reach widened globally during recent years (Nair et al. 2007; Powell et al. 2013). Scientists are currently investigating the conditions that might be fostering this spread and increase (Kaneko and Colwell 1975; Martinez-Urtaza et al. 2016) so that it might be halted, but in the meantime, early detection is important to seafood consumers in Europe, Asia and the US (Terzi Gulel and Martinez-Urtaza 2016).

*Vibrio parahaemolyticus* is the most prevalent of more than 30 Vibrio species reported and is among the 12 which are pathogenic (Skovgaard 2012). With an incubation period of about 15 h (ranging from 4 to 96 h), a dose of about 2 × 10^5^–3 × 10^7^ cfu is sufficient to lead to acute gastroenteritis (Costa Sobrinho et al. 2014; Ottaviani et al. 2012; Shimohata and Takahashi 2010; Vengadesh et al. 2014) and may be life-threatening for people with weak immune disorders, although the infection is often self-limited (Varnam and Evans 1991). Because *V. parahaemolyticus* is usually transmitted along the food supply chain through seafood (Caburlotto et al. 2016; Wong et al. 1999), it has the potential to further increase as the popularity of seafood as a source of healthy protein extends throughout the world (Zhang and Orth 2013). About 90% of global aquaculture products come from sources in the Asian region, particularly China, from where they are exported in massive quantities to overcome a scarcity in other countries (Liao and Chao 2009).

Common techniques used for the detection of *V. parahaemolyticus* include cultural (Shen et al. 2011), biochemical (Rosec et al. 2012), serological (Bisha et al. 2012), and immunological methods (Maniyankode et al. 2013). Requiring numerous analytical steps, all of these methods take up to a few days to provide a confirmed result. Apart from being laborious, the sensitivity of these methods needs to be improved as interference from other bacteria in the seafood samples, especially other *Vibrio* spp, can sometimes lead to a false result (Di Pinto et al. 2011). Thus, there is a real need to develop rapid methods and strategies for on-site *V. parahaemolyticus* monitoring.

Biosensing strategies are showing great promise with such features as being time-saving, cost-effective, practical, and able to perform real-time analysis (Fernandes et al. 2015; Hushiarian et al. 2015b; Lu et al. 2013; Tian et al. 2016; Zhang et al. 2013). In the last decade, electrochemical DNA biosensors have revolutionized modern analysis for detecting contaminants in a range of foods and environments (Celik et al. 2013; Dong et al. 2012; Dutse et al. 2013; Hushiarian et al. 2015a; Singh et al. 2013; Yin et al. 2013). Numerous electrochemical biosensors, based on screen-printing and with DNA immobilized on their surfaces, have been reported in the scientific literature (Alocilja et al. 2013; Das et al. 2014; Ding et al. 2012; Pal and Alocilja 2010; Paniel and Baudart 2013) and have been successfully employed for the fabrication of electrodes for mass production of disposable, low-cost devices (Caramit et al. 2015; Monteiro et al. 2015).

A number of attempts to develop portable biosensors for monitoring foodborne pathogens have been reported (Ferguson et al. 2011; Lee et al. 2016; Qin et al. 2016), but there would appear to be none which take this approach to detection of *V. parahaemolyticus*. Polylactide (PLA)—stabilized gold nanoparticles (AuNPs) have been widely used in a variety of analytical sensing applications (Han et al. 2014; Nordin et al. 2016; Song et al. 2006; Wu et al. 2011) and here gold nanoparticles (AuNPs) stabilized by the nanofiber were used to modify the electrode surface to increase the active surface area of the working electrode (WE) (Ding et al. 2012; Wu et al. 2011).

**Fig. 1:**
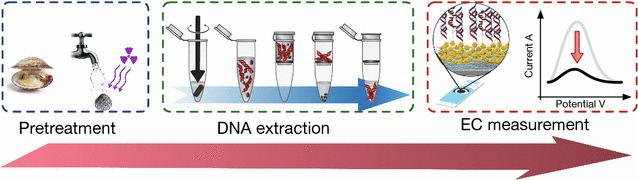
Schematic diagram depicting the steps in the process from pretreatment to electrochemical analysis

In summary, with the goal of developing an efficient in situ screening technique, a DNA hybridization-based portable biosensor labeled with methylene blue (MB) was customized. Figure 1 provides an overview of the simple process used. It begins with the pretreatment of the cockles prior to DNA extraction. The extracted DNA is then used as the sample for electrochemical (EC) analysis. The device has a selective probe designed for *V. parahaemolyticus* and is able to directly determine residues of this pathogen in extracted genomic DNA samples, without the need for previous cleanup or purification steps.

## Materials and methods

### Material and reagent

Gold (III) chloride trihydrate (Sigma-Aldrich) and poly (lactic acid) resin, commercial grade 4042D (NatureWorks) were of analytical grade and were used without further purification. Gold nanoparticles (AuNPs) and polylactic acid-stabilized gold nanoparticles (PLA-AuNPs) were synthesized and characterized, as described in detail in our previous report which formed the foundation for this improvement (Nordin et al. 2016). Electrochemical measurements were performed using a portable single-technique customized potentiostat (DropSens, Spain) for use with electrochemical sensor consisting of a screen printed electrode. The device contained a microprocessor, which controls the potential applied to the sensor and measures the current response. The instrument automatically converts current into a value through a calibration equation and is internally recorded and displayed on the LCD simultaneously. Conveniently, the device can be powered by a lithium ion battery and connected directly to a personal computer for data transfer. The sequences of ssDNA probe and complementary DNA were selected by exploring the National Center for Biotechnology Information (NCBI) database. Synthetic oligonucleotides (20-mer ssDNA probe, 20-mer complementary DNA, 20-mer mismatched DNA and 21-mer non-complementary DNA) were purchased (as lyophilized powder) from First BASE Laboratories, Malaysia with the following sequences: thiolated ssDNA probe: 5′-/5ThioMC6-D/CGGATTATGCAGAAGCACTG-3′, complementary DNA: 5′-CAGTGCTTCTGCATAATCCG-3′, one-base mismatched DNA: 5′-CAGTGCTTCTGC**Ṫ**TAATCCG-3′, three-base mismatched DNA: 5′-CAGTGCTTCT**ĊṪṪ**TAATCCG-3′ and non-complementary DNA: 5′-CGCACAAGGCTCGACGGCTGA-3′. The stock solutions of all oligonucleotide (100 µmol l^−1^) were prepared with sterile Tris–EDTA (TE) solution (10 mM Tris–HCl, 1 mM EDTA, pH 7.5) divided into analytical portions and kept at −4 °C. The appropriate dilutions were made as needed.

### Preparation of bacteria cell lysates

*Vibrio parahaemolyticus* ATCC 17802 as reference strains and nine other bacterial strains of common foodborne pathogen (*V. parahaemolyticus*, *C. jejuni*, *L. monocytogenes, S. Typhimurium, S. enteritidis, K. pneumonia*, *E. coli* O157:H7, *B. cereus* and *V. alginolyticus*) employed for electrochemical DNA biosensor validation were acquired from the Microbial Food Safety and Quality Laboratory, Universiti Putra Malaysia (UPM). We inoculated isolates into a growth broth with 20% glycerol and stored them at −60 °C. We then prepared fresh working culture as needed. We isolated genomic DNA from bacteria by a modified boiled lysis method (Ivanov and Bachvarov 1987) and determined its purity and quantity using an Eppendorf BioPhotometer D30 (Germany). We denatured the DNA in a Thermal Cycler at 92 °C for 2 min and rapidly cooled it in iced water prior to application in the biosensor. The DNA concentration and purity was determined using the biophotometer.

### Preparation of cockle samples

2 kg of cockles (*Anadara granosa*) freshly delivered that day were obtained from the wet market in Serdang, Selangor, Malaysia and quickly brought to the laboratory in an iced cooler box. For the study, the cockles were divided into two groups, namely spiked and unspiked group with the assumption that cockles are harvested uniformly from the beginning of harvesting until being placed in cold storage at the market. Half of the cockles in both groups were pre-treated by being stored at −20 °C for 24 h, followed by exposure to UV light at 20 °C for 4 h prior to DNA extraction. This pretreatment was considered as a preventive measure to limit or at least minimize naturally accumulated *V. parahaemolyticus* in the cockles. However, a higher pasteurization regime of 70 °C was not applied as the aim of the controlled condition in this study was to mimic the actual situation of fresh cockles. Meanwhile, the other half of the samples in both groups were directly sent for analysis as soon as the samples arrived in the laboratory.

### Treatment of cockle samples

Each cockle was washed in distilled water and scrubbed free of dirt before the tissue was removed from the shell using a sterile forcep in a laminar flow cabinet. About 10 g of cockle tissue sample was homogenized with homogenizer in 90 ml of sterile TSB (tryptic soy broth, 3% NaCl purchased from Merck, Malaysia) for 60 s. A known amount of *V. parahaemolyticus* inoculum was added to 9 ml of homogenized sample broth for the spiked samples while the unspiked samples were used as a negative control. Genomic DNA of the fresh cockles could be extracted from spiked and unspiked samples where the DNA concentration and purity could be determined using a biophotometer.

### Portable biosensor’s measurement procedure

The capacity and capability of the developed portable DNA biosensor was investigated by measurement in 0.1 M PBS (phosphate buffer saline pH 7 from Merck Malaysia) after the electrode was immersed in 20 µmol l^−1^ of MB for 30 min. For this work we used a three electrode carbon screen-printed system (Dropsens, DRP-550) consisting of a carbon working electrode (diameter 4 mm), a platinum counter electrode and a quasi-silver reference electrode. We immersed the electrode in 20 µM MB methylene blue from Merck Malaysia) for 30 min, washed it with 0.5 M PBS/20 mM NaCl (pH 4.5) and rinsed it with deionized water prior to measurement. The same procedure was applied for all interactions including probe DNA, complementary DNA, mismatched DNA and non-complementary DNA samples. We took the DPV measurements of the MB electrochemical reduction in the potential range from −0.5 to 0.25 V at the step potential of 0.005 V and the modulation amplitude of 0.05 V with the scan rate of 7.73 mVs^−1^ in 0.1 M PBS (pH 7) containing no indicator. We subsequently studied the sensitivity and reproducibility of the customized portable DNA biosensor. Validation studies of the portable DNA biosensor using bacteria cell lysates and fresh cockles were further conducted. All reported results were the measurement of the mean value from three replicates. We investigated hybridization between probe and synthetic oligonucleotides by DPV using a µAutolab III (Eco-chemie, Netherland) voltammetric analyser together with General Purpose Electrochemical System (GPES 4.9) software. We found significant characteristics of PLA-AuNPs as modifier from preliminary study, which demonstrated good sensitivity, stability, reproducibility, and repeatability. We used SPCE modified with PLA-AuNPs, denoted as SPCE\PLA-AuNPs for this study. A drop casting method was used for the DNA immobilization and hybridization. We immobilized a 25 µl of thiolated ssDNA probe (1.2 µM) on the SPCE\PLA-AuNPs and air dried it for 24 h at room temperature. Then we pipetted 25 µl of different concentrations of complementary DNA on to the SPCE\PLA-AuNPs\ssDNA for 40 min at room temperature. We studied the surface morphology of SPCE prior to modification using scanning electron microscopy (SEM, JEOL JSM 6400). We conducted further studies of hybridization time and temperature using the optimum concentration of complementary DNA.

## Results

The selectivity of the optimized DNA biosensor was assessed by measuring its responses towards different gene sequences related to *V. parahaemolyticus*. The signals measured were as shown in Table 1. After the hybridization of the target DNA, the sensor showed the lowest oxidation signals with peak currents of 1.00 μA. The oxidation signal was about 2.70 times lower than that of the bare electrode. The hybridization with the non-complementary sequence also show that the peak current was much higher than that obtained from the hybridization of the target DNA.

**Table 1:** Selectivity of MB peak current in 0.1 mol l^−1^ PBS (pH 7) at scan rates of 0.1 V s^−1^ after 30 min incubation in 20 µM MB

Sample	i_pa_ (× 10^−6^ A)	Selectivity rate (%)
Bare electrode	3.63	–
Non-complementary DNA	2.73	75.21
3-Base mismatched DNA	1.83	50.41
1-Base mismatched DNA	1.40	38.57
Complementary DNA	1.00	27.55

The percentage of selectivity rate was then calculated based on the following equation: Selectivity rate (%) = (A_t_/A_0_) × 100, where A_0_ is the mean MB peak current obtained (n = 3) without hybridization and A_t_ is the mean MB peak current obtained (n = 3) with different types of hybridization i.e. non-complementary DNA, 3-base mismatched DNA, 1-base mismatched DNA and complementary DNA. When the ssDNA molecule was used as the capture probe, the hybridization reaction was recorded through the decreases in current signals after the duplex formation on the electrode surface.

### Reproducibility

To investigate the reproducibility and precision of the optimized DNA bio- sensor, we used a freshly prepared biosensor to detect 0.2 μM target DNA.

### Sensitivity

**Fig. 2:**
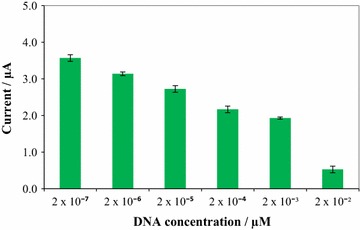
Histogram of effect of different DNA concentration on peak current

**Fig. 3:**
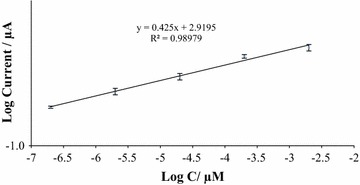
The plot of reduction peak current of MB against log concentration of DNA

The developed electrochemical DNA biosensor was then studied, using the immobilized ssDNA to hybridize with various concentrations of the target DNA of *V. parahaemolyticus* as shown in Fig. 2. This device was able to detect target DNA in concentrations ranging from 2 × 10^−7^ to 2 × 10^−2^ µM with a linear regression coefficient of 0.989 (Fig. 3).

### Cross-reactivity study

**Fig. 4:**
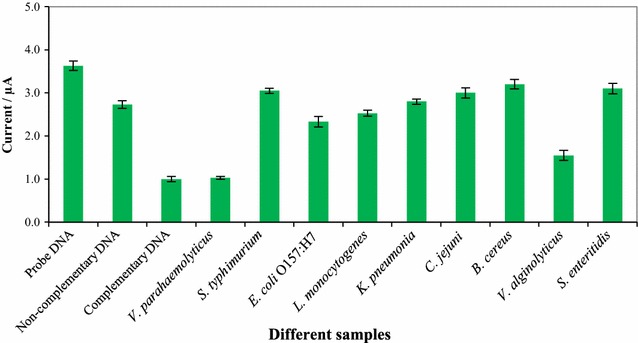
Cross reactivity study of portable DNA biosensor against various foodborne pathogens

Figure 4 shows the results of the cross-reactivity study conducted with the portable DNA biosensor in the presence of various foodborne pathogens which mimicked the environment of this food sample. From these results, it can be seen that the intensity of the oxidation current decreased in the order of *V. parahaemolyticus* < *V. alginolyticus* < *E. coli* O157:H7 < *L. monocytogenes* < *K. pneumonia* < *S.* Typhimurium < *C. jejuni* < *S. enteritidis* < *B. cereus*.

### Evaluation of fresh cockles

Finally, the method trueness of the DNA-based sensing system was evaluated by monitoring real samples (fresh cockles) with the referenced polymerase chain reaction (PCR). It successfully distinguished the available *V. parahaemolyticus* in the treated group (spiked and unspiked) samples. These results were in positive correlation with PCR results previously discussed.

When unspiked sample of cockles were hybridized at the SPCE/PLA-AuNPs/ssDNA probe, no significant variation was observed among the samples. These results indicate that peak currents were less varied within a sample of cockles regardless of whether they were treated or not.

**Fig. 5:**
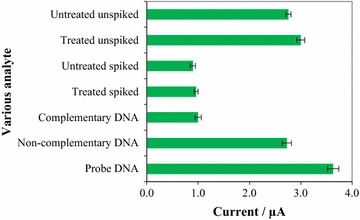
Detection of *V. parahaemolyticus* in fresh cockle samples using portable DNA biosensor

The optimized biosensor was validated using treated cockle samples (spiked and unspiked with *V. parahaemolyticus* culture cell) and untreated cockle samples (spiked and unspiked with *V. parahaemolyticus* culture cell). The lowest peak current was observed when the target DNA from spiked (treated and untreated cockle samples) was detected. Conversely, unspiked (treated and untreated cockle samples) produced higher current, which was similar to that of the non-complementary DNA signal (Fig. 5).

**Fig. 6:**
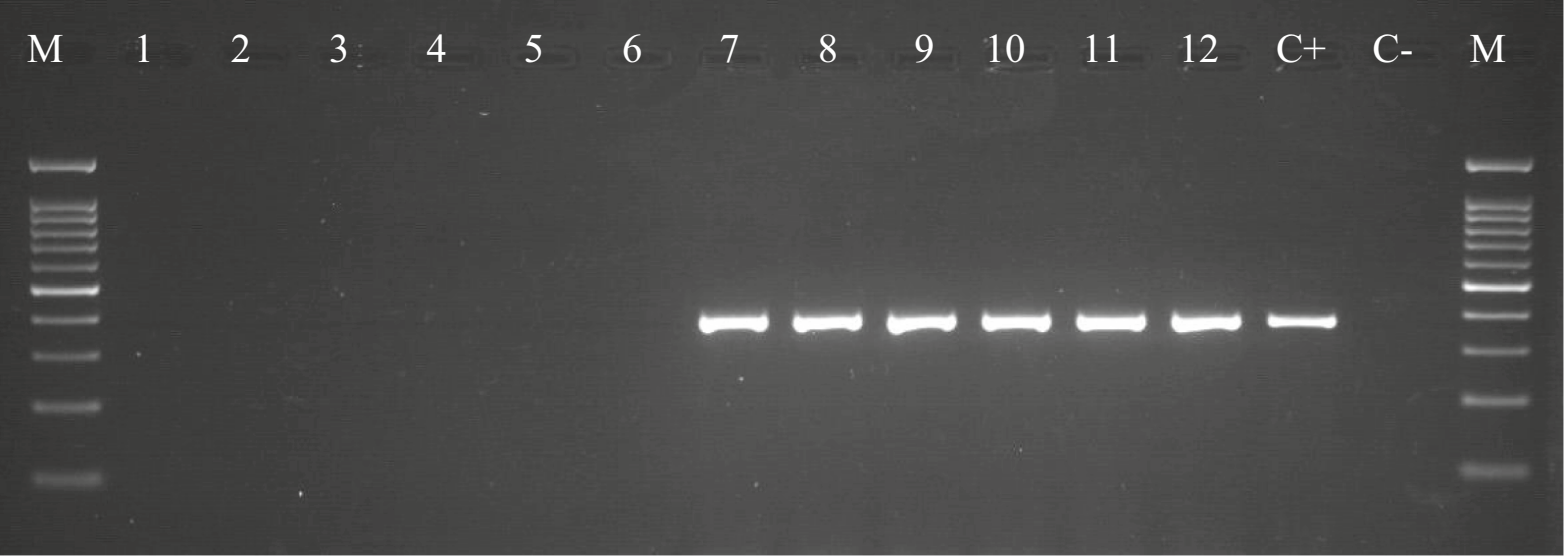
Polymerase chain reaction results of fresh cockle samples (Lane M: 2 μl of 100 bp DNA ladder, *Lane 1*–*3* untreated unspiked samples, *Lane 4*–*6* treated unspiked samples, *Lane 7*–*9* untreated spiked samples, *Lane 10*–*12* treated spiked samples, *Lane C*+ positive control (*V. parahaemolyticus* toxR gene), *Lane C*− negative control (sterile distilled water)

With this in mind, we used gel electrophoresis to validate the presence of *V. parahaemolyticus* in fresh cockle samples. From PCR analysis, *V. parahaemolyticus* were found in spiked (treated and untreated cockle samples) as shown by Lane 7, 8, 9, 10, 11, and 12, whereas unspiked (treated and untreated cockle samples) at Lane 1, 2, 3, 4, 5 and 6 were devoid of *V. parahaemolyticus* occurrence (Fig. 6). Confirmations of *V. parahaemolyticus* present in the spiked (treated and untreated cockle samples) by PCR (The appeared band in agarose gel electrophoresis at 368 bp) correlate with the quantity of colony as discussed previously. However, the PCR results showed no detection of *V. parahaemolyticus* in unspiked (treated and untreated cockle samples) even though the amount of colony indicated presence of *V. parahemolyticus* in the samples.

## Discussion

The *Vibrio parahaemolyticus* pathogen is an important seafood-borne enteropathogen that continues to cause acute human gastroenteritis throughout the world (Zhang et al. 2016) and its rapid detection is of great significance for food safety and disease diagnosis (Cheng et al. 2016).

Although most conventional DNA-based biosensors have the advantage of high selectivity due to the unique structure of the probe DNA, the complicated preparation process and the low electrochemical signal intensity discourage many attempts at practical application (Wang et al. 2016). Additionally, many efforts suffer from one or more other drawbacks including long analytical time, high analytical costs and expensive instrumentation (Sha et al. 2016).

Recent examples which show promise include a highly selective and sensitive SERS-based aptasensor which used Au@Ag core–shell nanoparticles as the active substrate (Duan et al. 2016) which needs to be more sensitive and a label-free electrochemiluminescence (ECL) immunosensor based on multi-functionalized graphene oxide but which needs to be tested in the field (Sha et al. 2016).

In the work presented here, the above-mentioned disadvantages were anticipated and overcome. A previously constructed electrochemical mechanism (Nordin et al. 2016) was modified and a simplified approach for fabrication of the DNA biosensor was based on a screen-printed carbon electrode reader with polylactide—stabilised gold nanoparticles and methylene blue as the redox indicator. Results for the analytical parameters of this sensor (selectivity, reproducibility, sensitivity and cross-reactivity) compare favourably with results from a range of other DNA sensors as recently reviewed by Wang et al. (2016).

The percentage of selectivity rate of the optimized sensor was significantly high. After the hybridization of the target DNA, it showed the lowest oxidation signals with peak currents of 1.00 μA. The oxidation signal was about 2.70 times lower than that of the bare electrode. The hybridization with the non-complementary sequence also showed that the peak current was much higher than that obtained from the hybridization of the target DNA. When the ssDNA molecule was used as the capture probe, the hybridization reaction was recorded through the decreases in current signals after the duplex formation on the electrode surface. For 1-base mismatched and 3-base mismatched DNA, the oxidation current increased by 40 and 38%, respectively, in comparison to that of complementary DNA which suggests that the hybridization was weak. The results indicate limited interaction occurred between MB and guanine bases as only a small amount of MB was available on the surface with hybridized dsDNA.

Its reproducibility capacity plays an extremely important role in practical application for a biosensor. With this device, the relative standard deviation (RSD) toward the target DNA concentration of 4.33% (n = 6) was an excellent result in terms of the reproducibility and precision of the optimized DNA biosensor and compares well with other attempts such as that of Sha et al. (2016) who reported RSD measurements of 7.8%.

Additionally, the device demonstrated a low detection limit, calculated to be 2.16 × 10^−6^ µM (3 σ/m, and a wide linear range for the target DNA sequence being analyzed. The optimized electrochemical DNA biosensor was able to reach a lower quantitation limit (10 σ/m) of 7.2 × 10^−6^ µM for DPV than had previously been reported (Sha et al. 2016; Zhao et al. 2007).

In the presence of various foodborne pathogens, our portable sensor was successfully able to detect *V. parahaemolyticus*. After hybridization of the SPCE/PLA-AuNPs/ssDNA with target DNA, the peak current decreased greatly, which suggests that dsDNA was formed at the modified electrode surface. No significant decrement in peak current was observed after the probe DNA was hybridized with non-*V. parahaemolyticus* target DNA, indicating that it was poorly hybridized. Pleasingly, the optimized DNA biosensor was clearly highly specific because non-*V. parahaemolyticus* isolates of crude DNA fragments did not show a significant enhancement in peak currents compared with *V. parahaemolyticus* isolates.

Finally, when the method trueness of the DNA-based sensing system was evaluated by monitoring real samples (fresh cockles) with the referenced polymerase chain reaction (PCR), it was able to successfully distinguish the available *V. parahaemolyticus* in the treated group (spiked and unspiked) samples. When unspiked sample of cockles were hybridized at the SPCE/PLA-AuNPs/ssDNA probe, no significant variation was observed among the samples indicating that peak currents were less varied within a sample of cockles regardless of whether they were treated or not. The results suggest that the optimized DNA biosensor is highly selective towards *V. parahaemolyticus* compared with non-*V. parahaemolyticus* target DNA. When the optimized biosensor was ultimately validated, the lowest peak current was observed when the target DNA from spiked (treated and untreated cockle samples) was detected while conversely, unspiked (treated and untreated cockle samples) produced higher current, which was similar to that of the non-complementary DNA signal, probably due to the inevitable non-specific binding formed during the hybridization steps. When gel electrophoresis was subsequently tried to validate the presence of *V. parahaemolyticus* in fresh cockle samples, the PCR analysis found *V. parahaemolyticus* in spiked (treated and untreated cockle samples) whereas unspiked (treated and untreated cockle samples) were devoid of *V. parahaemolyticus* occurrence, correlating with the quantity of colony as discussed previously. Interestingly, the PCR results showed no detection of *V. parahaemolyticus* in unspiked (treated and untreated cockle samples) even though the amount of colony indicated the presence of *V. parahemolyticus* in the samples. He et al. (2011) suggest that this might be due to low purity with high contamination of extracted DNA such as protein (He et al. 2011).

In summary, the optimized portable electrochemical DNA biosensor described here was able to detect the presence of *V. parahaemolyticus* in fresh cockle samples even when there were other bacteria present. Thus, not only is this simple device able to discriminate between contaminated and uncontaminated samples, it is also superior to other current detection methods because it dramatically reduces the number of analyses which need to be performed. Its reliable sensitivity together with its portability make it potentially suitable for providing convenient on-site monitoring and analysis of *V. parahaemolyticus* in real samples.

## Abbreviations

AuNP: gold nanoparticle
DPV: differential pulse voltammetry
EC: electrochemical
ECL: electrochemiluminescence
MB: methylene blue
NCBI: National Center for Biotechnology Information
PCR: polymerase chain reaction
PLA: poly lactic acid
RSD: relative standard deviation
SPCE: screen printed carbon electrode
UPM: Universiti Putra Malaysia
WE: working electrode
